# Effects of physical training combined with transcranial direct current stimulation on maximal strength and lower limb explosive strength in healthy adults

**DOI:** 10.3389/fspor.2024.1446588

**Published:** 2024-09-20

**Authors:** Jintong Liu, Chunlei Li, Junhui Fang, Haokai Xu, Xingyue Zhang, Fan Zhao

**Affiliations:** ^1^ School of Strength and Conditioning Training, Beijing Sport University, Beijing, China; ^2^ Sport Training Center, China Institute of Sport Science, Beijing, China; ^3^ Sports Coaching College, Beijing Sport University, Beijing, China; ^4^ Competitive Sports Department, Beijing Research Institute of Sports Science, Beijing, China

**Keywords:** transcranial direct current stimulation, explosive strength, muscle strength, physical training, cerebral cortex excitability

## Abstract

**Objective:**

The purpose of this systematic review and meta-analysis was to investigates whether transcranial direct current stimulation applied during physical training increases muscle strength in comparison with sham tDCS combined with physical training.

**Methods:**

Randomized controlled trials of the effects of tDCS combined physical training intervention on muscle strength and cortical excitability were collected by searching Web of Science, Pubmed, EBSCO, CNKI. The retrieval date ends in April 2024. 11 randomized controlled trials are finally included. The total sample size of the study is 338. The experimental group was subjected to tDCS combined with physical training intervention, and the control group was physical training combined with sham tDCS intervention.

**Results:**

There is a significant increase in maximal strength (SMD = 0.38; 95% CI: 0.09, 0.67; *p *= 0.01) and lower limb explosive strength (MD = 2.90; 95% CI: 1.06, 4.74; *p *= 0.002) when physical training was performed with tDCS, but not following physical training combined with sham tDCS. Subgroup analysis of the subject population showed an increase in muscle strength in those with training experience following tDCS combined with physical training (SMD = 0.39; 95% CI: 0.08, 0.70; *p *= 0.01), but not for those without training experience (SMD = 0.29; 95% CI: −0.06, 0.63; *p *= 0.10). Motor evoked potential (MEP) wave amplitude increased significantly following physical training with tDCS (SMD = 0.71; 95% CI: 0.18, 1.24; *p *= 0.008), but was not different between groups (SMD = 0.16; 95% CI: −0.33, 0.65; *p *= 0.52).

**Conclusions:**

tDCS combined with physical training intervention can improve muscle strength, lower limb explosive strength and cerebral cortex excitability. Compared to tDCS combined with training of small muscle groups, tDCS combined with training of large muscle groups was more effective in improving muscle strength. Muscle strength was more likely to improve after tDCS combined with physical training in people with physical training experience compared with people without physical training experience. The combination of tDCS with physical training intervention and the sham-tDCS with physical training intervention both increased cortical excitability.

**Systematic Review Registration:**

https://www.crd.york.ac.uk/, PROSPERO, identifier (CRD42024550454).

## 1 Introduction

Muscle strength represents a critical determinant of athletic performance, with a complex interplay between myogenic and neurogenic factors influencing its development (1). In daily training sessions, coaches mostly start from the myogenic factors, and produce adaptive changes in the skeletal muscle system through appropriate training means (2, 3). From a neurological perspective, cortical excitability is also one of the most important factors affecting muscle strength and plays a pivotal role in the generation of muscle power (4). Neuropriming techniques use neurons as an entry point to increase cortical excitability through stimulation of the brain nerves, strengthening the connection between the brain and the muscles, and thus improving muscle strength (5, 6). Transcranial direct current stimulation (tDCS), as one of the Neuropriming techniques, is a non-invasive brain stimulation technique that generates weak direct current through electrodes placed in the skull (7). It was initially applied in the medical field to treat diseases such as Parkinson's and stroke (8, 9). Given that tDCS has been demonstrated to enhance patients’ motor ability (10–12), as well as the characteristics of portability, noninvasiveness, and easy manipulation (13), it has been used by many researches to explore the stimulation effects on health people and athletes’ performance.

With long-term physical training, the skeletal muscle system may be slow to improve strength due to a ceiling-like effect, so some researchers, in order to explore more effective ways to improve muscle strength, have applied tDCS to physical training, thus exploring whether physical training combined with tDCS can effectively promote muscle strength improvement. However, the effect of brain stimulation combined with physical training as a novel approach to enhancing performance is contingent upon the dosage of tDCS, individual differences, and the training protocol employed. Therefore, findings on the effectiveness of tDCS in combination with physical training have been inconsistent. Hendy et al. (14) indicated that maximum strength of the untrained biceps brachii and cerebral cortex excitability were increased following 2 weeks of physical training combined with 2 mA tDCS. Other studies have also shown that knee extension (15), wrist extension (16), and knee flexion (17) strengths were improved after a period of tDCS combined with physical training intervention compared to the physical training with sham tDCS. However, some studies have shown that the tDCS combined physical training intervention did not improve muscle strength (18), and cortical excitability (19, 20). In conclusion, there is still some controversy regarding the effects of tDCS combined with physical training intervention on muscle strength and cortical excitability, so it is necessary to integrate and analyse this type of research.

The published studies reviews on tDCS have focused exclusively on the effects of single tDCS session on muscle strength in healthy populations. There is, however, a notable absence of reviews and evaluations of the effects of tDCS combined with physical training as concurrent tDCS and training on physical performance (21, 22). Therefore, the purpose of this systematic review and meta-analysis was to investigates transcranial direct current stimulation applied during physical training increases muscle strength in comparison with sham tDCS combined with physical training.

## 2 Methods

### 2.1 Search strategy

Keywords used in the search were as follows: (“Transcranial Direct Current Stimulation” OR “tDCS” OR “Transcranial Electrical Stimulations” OR “Transcranial Electrical Stimulation”) AND (“Muscle Strength” OR “Strength Training” OR “physical training” OR “resistance training” OR “strength exercise”). Databases searched included PubMed, EBSCO, CNKI, and Web of Science. The deadline for searching was 18 April 2024. Figure 1 shows the screening process for studies. Taking the web of science database as an example, the specific search strategy is as follows:

#1 “transcranial direct current stimulation"[MeSH Terms] OR (“transcranial"[All Fields] AND “direct"[All Fields] AND “current"[All Fields] AND “stimulation"[All Fields]) OR “transcranial direct current stimulation"[All Fields] OR (“transcranial direct current stimulation"[MeSH Terms] OR (“transcranial"[All Fields] AND “direct"[All Fields] AND “current"[All Fields] AND “stimulation"[All Fields]) OR “transcranial direct current stimulation"[All Fields] OR “tdcs"[All Fields]) OR (“transcranial direct current stimulation"[MeSH Terms] OR (“transcranial"[All Fields] AND “direct"[All Fields] AND “current"[All Fields] AND “stimulation"[All Fields]) OR “transcranial direct current stimulation"[All Fields] OR (“transcranial"[All Fields] AND “electrical"[All Fields] AND “stimulations"[All Fields]) OR “transcranial electrical stimulations"[All Fields]) OR (“transcranial direct current stimulation"[MeSH Terms] OR (“transcranial"[All Fields] AND “direct"[All Fields] AND “current"[All Fields] AND “stimulation"[All Fields]) OR “transcranial direct current stimulation"[All Fields] OR (“transcranial"[All Fields] AND “electrical"[All Fields] AND “stimulation"[All Fields]) OR “transcranial electrical stimulation"[All Fields])
#2 “muscle strength"[MeSH Terms] OR (“muscle"[All Fields] AND “strength"[All Fields]) OR “muscle strength"[All Fields] OR (“resistance training"[MeSH Terms] OR (“resistance"[All Fields] AND “training"[All Fields]) OR “resistance training"[All Fields] OR (“strength"[All Fields] AND “training"[All Fields]) OR “strength training"[All Fields]) OR ((“physical examination"[MeSH Terms] OR (“physical"[All Fields] AND “examination"[All Fields]) OR “physical examination"[All Fields] OR “physical"[All Fields] OR “physically"[All Fields] OR “physicals"[All Fields]) AND (“education"[MeSH Subheading] OR “education"[All Fields] OR “training"[All Fields] OR “education"[MeSH Terms] OR “train"[All Fields] OR “train s"[All Fields] OR “trained"[All Fields] OR “training s"[All Fields] OR “trainings"[All Fields] OR “trains"[All Fields])) OR (“resistance training"[MeSH Terms] OR (“resistance"[All Fields] AND “training"[All Fields]) OR “resistance training"[All Fields]) OR ((“strength"[All Fields] OR “strengths"[All Fields]) AND (“exercise"[MeSH Terms] OR “exercise"[All Fields] OR “exercises"[All Fields] OR “exercise therapy"[MeSH Terms] OR (“exercise"[All Fields] AND “therapy"[All Fields]) OR “exercise therapy"[All Fields] OR “exercising"[All Fields] OR “exercise s"[All Fields] OR “exercised"[All Fields] OR “exerciser"[All Fields] OR “exercisers"[All Fields]))
#3 (#1 AND #2)

**Figure 1:**
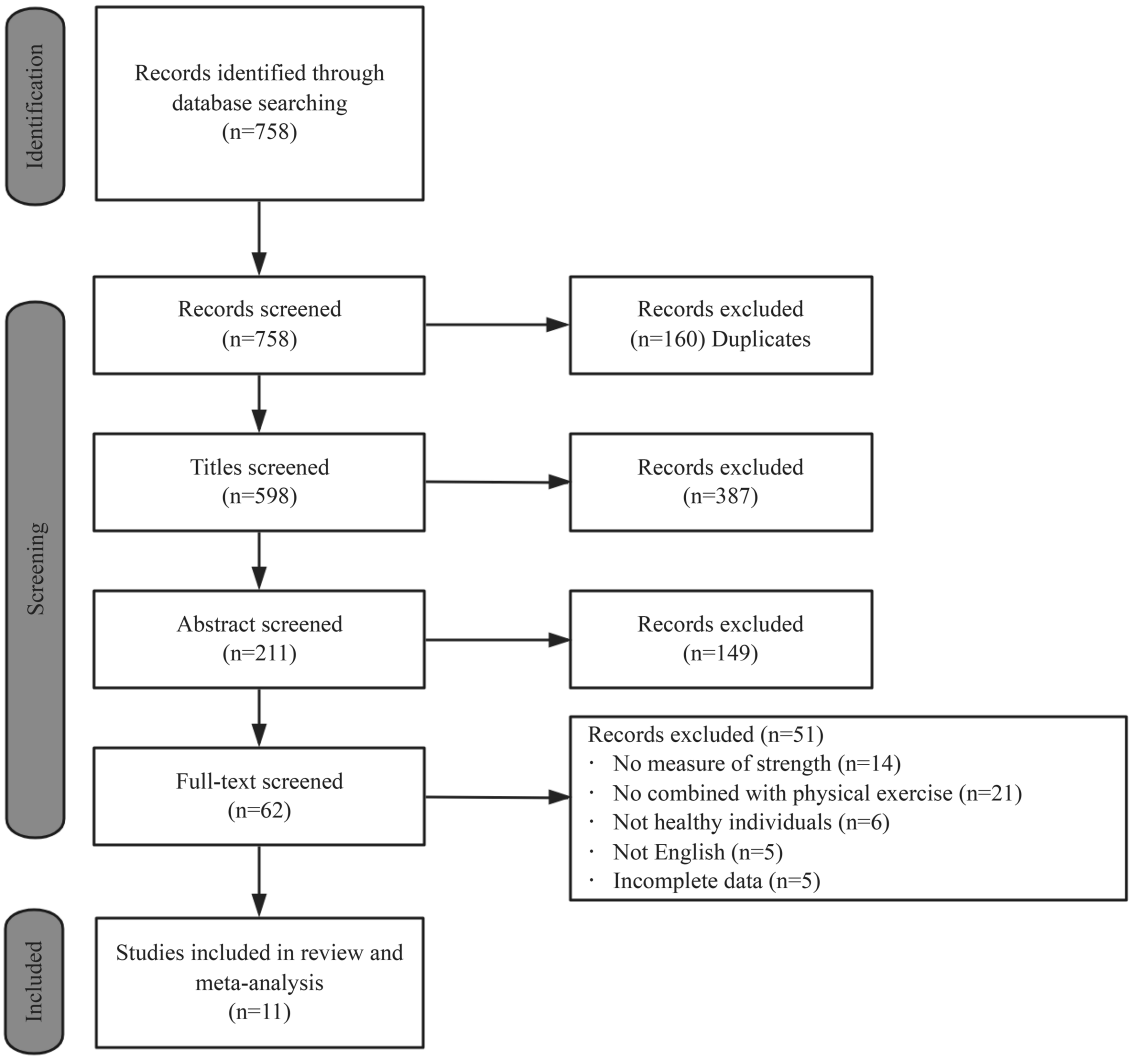
Flow chart of the selection of studies.

### 2.2 Inclusion and exclusion criteria

Determination of study inclusion criteria based on the PICOS principle (23). Studies were included for review if they fulfilled the following selection criteria: (a) Intervention and control measures: experimental group received tDCS stimulation and training intervention, control group received sham tDCS plus training intervention; (b) Outcome measures: metrics related to maximal strength and explosive strength, measures related to cortical excitability (motor-evoked potentials); (c) Study design: the included studies were randomized controlled trials or self-controlled experiments.

The exclusion criteria were as follows: (a) Participants were patients, the elderly, or athletes; (b) No experimental data; (c) Repeatedly published literature; and (d) animal experiments.

### 2.3 Data extraction

The following data, presented as Microsoft Word files (Microsoft Corp.), were extracted from the included studies: (a) leader author, year of publication; (b) descriptive information of the sample including sample size and age; (c) tDCS protocols: electrode positioning and dosage; (d) training protocols: duration, intensity and frequency; (d) the methodology of strength and MEP assessment. Data extraction was carried out by 3 authors (Liu Jintong, Zhao Fan and Li Chunlei) and was cross-checked between them, with any discrepancies resolved by discussion and consensus.

### 2.4 Quality assessment

The Cochrane Risk Assessment Tool for assessing risk of bias was used to assess the risk of bias of the included randomized clinical trials. Assessment with this tool includes six domains: selection bias, performance bias, detection bias, attrition bias, reporting bias, and other bias. Each domain can be scored as: low risk of bias, some concerns, or high risk of bias. The study search, data extraction and quality assessment were carried out by two independent reviewers (Liu and Li), with a third reviewer involved in case of disagreement (Zhao).

### 2.5 Statistical analyses

This meta-analysis compared the effects of sham tDCS combined with physical training vs. tDCS in combination with physical training on maximal muscular strength, vertical jump and cortical excitability. Since the muscle strength and cortical excitability performance data in this study were continuous variables with inconsistent testing units, standardized mean difference (SMD) was used, whereas the vertical jump performance data were consistent with the testing units and were continuous variables, so they were expressed by mean difference (MD). Effect sizes were defined by the Cochrane Handbook, with SMD(MD) < 0.5 as a small effect size, 0.5 ≤ SMD(MD) < 0.8 as a medium effect size, and SMD(MD) ≥ 0.8 as a large effect size (24). Heterogeneity was explored using the *I*^2^ statistic. When *I*^2^* *= 0, it indicated that there was no heterogeneity between studies, when *I*^2^* *< 50%, it indicated that there was a low degree of heterogeneity between studies, and at this time, a fixed effect model was selected for analysis, when *I*^2^* *≥ 50%, a random effect model needed to be selected for analysis. Publication bias was visually assessed by creating funnel plots using Review Manager version 5.4 and by conducting Egger's regression tests on the results of studies that included 10 or more studies using Stata version 16.0. Statistical significance was determined at *p* < 0.05. Review Manager 5.4 was used for all analyses.

## 3 Results

### 3.1 Search results

758 studies were found after searching and 598 relevant studies were obtained after removing duplicate studies in EndNote X9.3.3 software. 62 studies were selected after reading the titles and abstracts, which were rescreened by reading the full text. Only 11 studies were included in this systematic review and meta-analysis after rereading the full texts (Figure 1).

### 3.2 Study characteristics

The included studies were published between 2013 and 2024; the participants included in the study were all healthy (age: 23.55 ± 2.54), with a total sample size of 338, totaling 166 in the experimental group and 172 in the control group. The control group was physical training intervention only and the experimental group was physical training combined with tDCS intervention. Information on the training program, the dosage of the tDCS were also included in the study. Table 1 shows the characteristics of the included studies.

**Table 1:** The basic information of the included studies.

Authors	Numbers	Age (years)	tDCS dosage					Training protocol	Main outcomes	Results
			Position of anodal electrodes	Position of cathode electrodes	Current dosage	Duration of stimulation	Size of electrode montage			
Hendy and Kidgell (25)	30	21–28	M1	Right supraorbital area	2 mA	20 min	25 cm^2^	4 × 6–8 repetitions of 70%1RM wrist extensors with a weighted dumbbell	Wrist extensors 1RM	–
Jung et al. (15)	55	19–65	M1	Supraorbital zone	2 mA	20 min	36 cm^2^	Compound physical training (strength, endurance, explosive strength) lasts 40 min.	Maximal isometric strength of elbow flexion	↑
									Maximal isometric strength of knee extension	↑
									Sargent jump	↑
Xiao et al. (26)	30	20–23	M1	–	2 mA	20 min	3.14 cm^2^	Foot core exercise, which consisted of foot doming, towel curls, toe spread and squeeze, and balance board training	Toe flexor strength	↑
Maeda et al. (17)	24	23.7 ± 1.3	M1	Ipsilateral upper arm	2 mA	10 min	25 cm^2^	3 sets of 10 maximum isokinetic knee flexors and knee extensors eccentric contractions at 30°/s; 150 s rest period between sets	Maximal knee extensor	↑
									Flexor torques	↑
Summers et al. (19)	14	28.8 ± 10.5	Cerebellum	Buccina muscle ipsilateral to the training hand	2 mA	30 min	70 cm^2^	An index finger extension and flexion tracking training program	MEP	–
Hendy and Kidgell (16)	10	25.9 ± 1.37	M1	Left supraorbital area	2 mA	20 min	25 cm^2^	4 × 6 repetitions of 70%1RM wrist extensors with a weighted dumbbell; 3 min recovery period between sets	Right wrist extensors 1RM,	–
									MEP	↑
Kim and Ko (27)	44	28.3 ± 2.8	M1	Right forehead	2 mA	20 min	25 cm^2^	30 s × 50%MVC grip exercise	MEP	
Hendy et al. (14, 28)	24	25.8 ± 2.9	M1	Left supraorbital area	1.5 mA	15 min	25 cm^2^	4 × 6 repetitions of 80%1RM bicep curls with a weighted dumbbell; 3 min recovery period between sets	Bicep curls 1RM	–
									MEP	↑
Gua (31)	36	18–21	M1	Ipsilateral shoulder	2 mA	20 min	25 cm^2^	5 × 5 repetitions of 85%1RM bulgarian split squat; 3–4 min recovery period between sets	Vertical jump touch height,	↑
									Squat 1RM	↑
Wu (29)	32	18–19	M1	Left supraorbital	2 mA	20 min	–	4 × (30–15–15–15) repetitions of 30%1RM loaded jump squat; 30 s recovery period between sets	Squat 1RM	↑
									Countermovement jump	↑
Ni (30)	33	19–21	M1	Ipsilateral shoulder	2 mA	20 min	25 cm^2^	5 × 5 repetitions of 85%1RM squat; 2–3 min recovery period between sets	Squat 1RM	↑
									Vertical jump height	↑

M1, primary motor cortex; MEP, motor evoked potential; “↑”, a significant improvement; “–”, no improvement.

### 3.3 Methodological quality and publication bias

**Figure 2:**
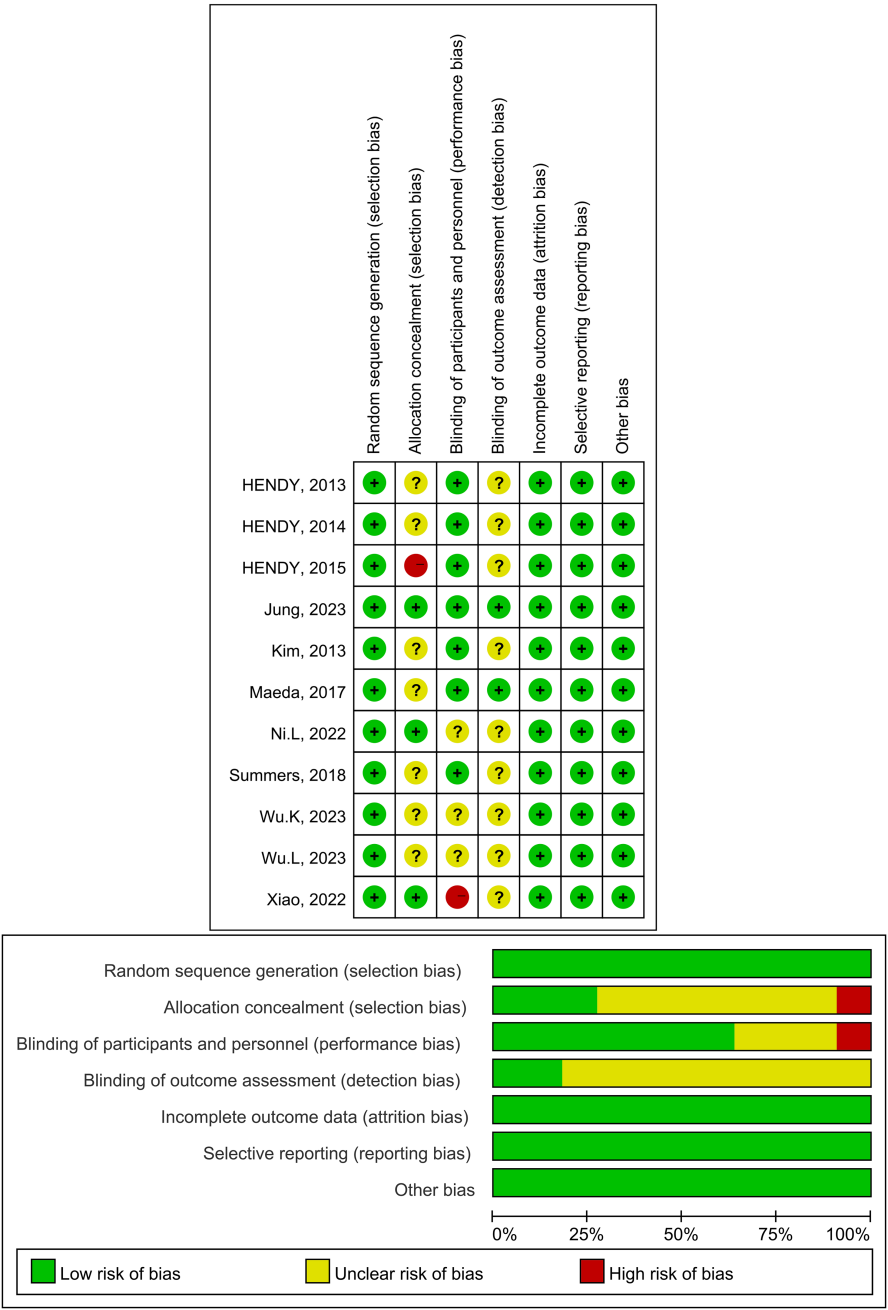
Risk diagram of the risk-of-bias tool for randomized trials.

A total of 11 papers were included, of which 11 described the randomisation methodology, 3 described the hidden allocation scheme, 7 described the specific blinding methodology, and 2 described the blinding methodology for outcome assessment. The data results were complete for all the included literature. In summary, 7 articles were of high quality, 2 articles were rated as medium risk, and 2 articles were rated as high risk. The results were presented in a traffic light diagram (Figure 2).

**Figure 3:**
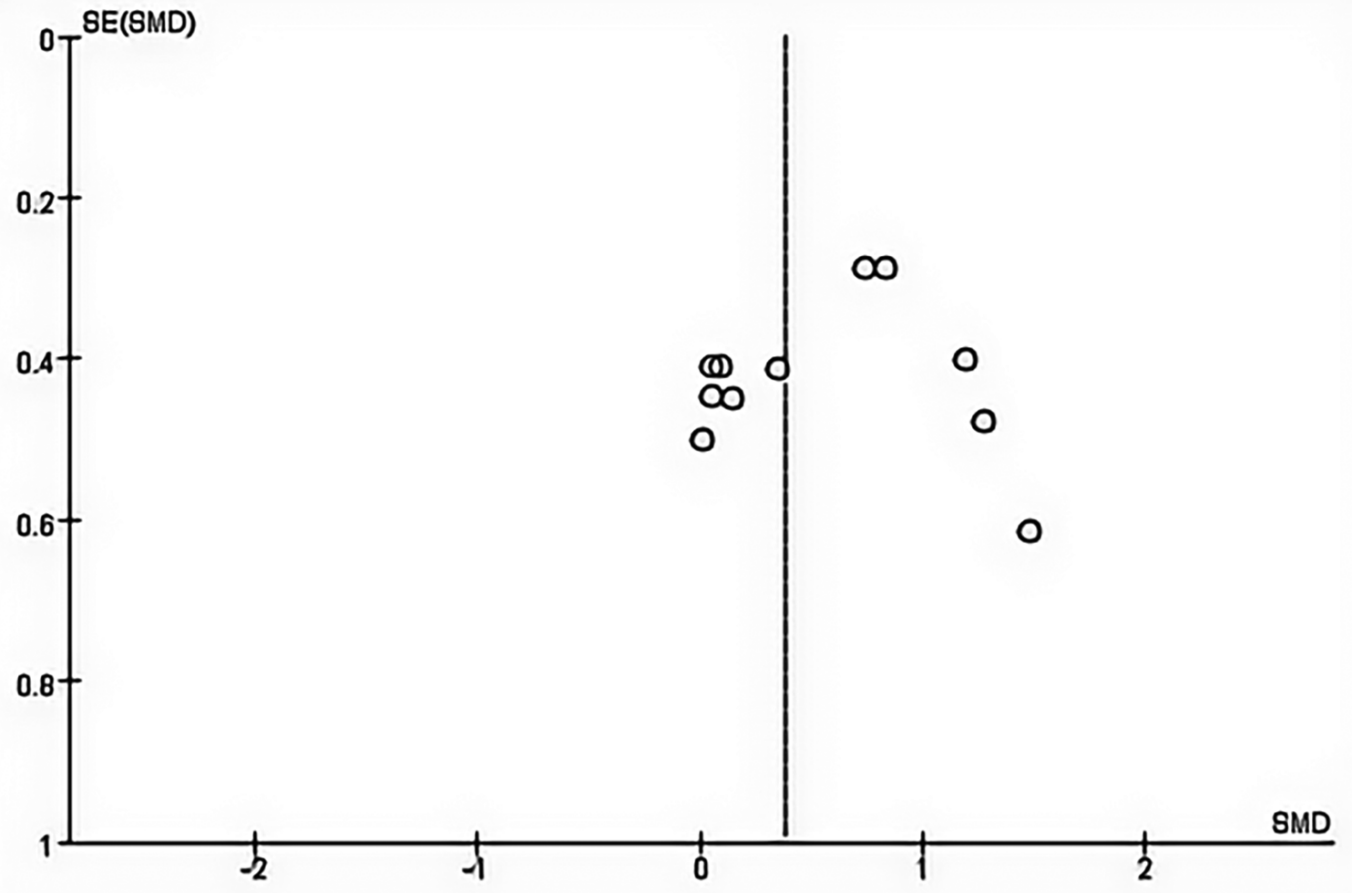
Begg's funnel plot of the maximal strength with 95% confidence interval.

Considering that there must be at least ten studies to assess publication bias, publication bias could not be assessed for lower limb explosive strength and cortical excitability due to the small number of studies (≤10). In addition, no evidence of publication bias was observed through visual evaluation of funnel plots and Egger's test results for maximal strength (*p* = 0.362) (Figure 3).

### 3.4 Meta-analysis results

#### 3.4.1 Maximal strength

**Figure 4:**
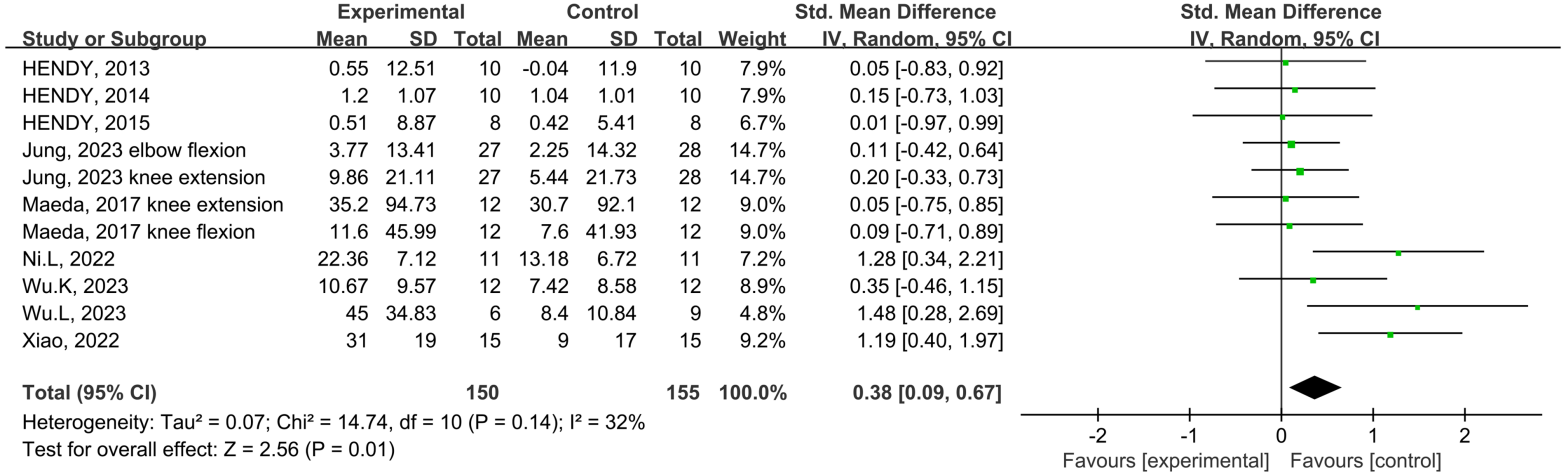
Meta-analyses of the effects of tDCS combined with physical training interventions on muscular strength.

Eight studies from the included studies reported the effects of tDCS in combination with physical training intervention on muscle strength. As the test for heterogeneity showed low heterogeneity (*p *= 0.14, *I*^2^ = 32%), and *I*^2^ < 50%, we used the fixed-effects model. Based on the meta-analysis, tDCS combined with physical training intervention was significantly more effective than physical training intervention alone in improving muscle strength, and the improvement of muscle strength reached the level of small effect size (SMD = 0.38, 95% CI: 0.09, 0.67, *p *= 0.01) (Figure 4).

**Figure 5:**
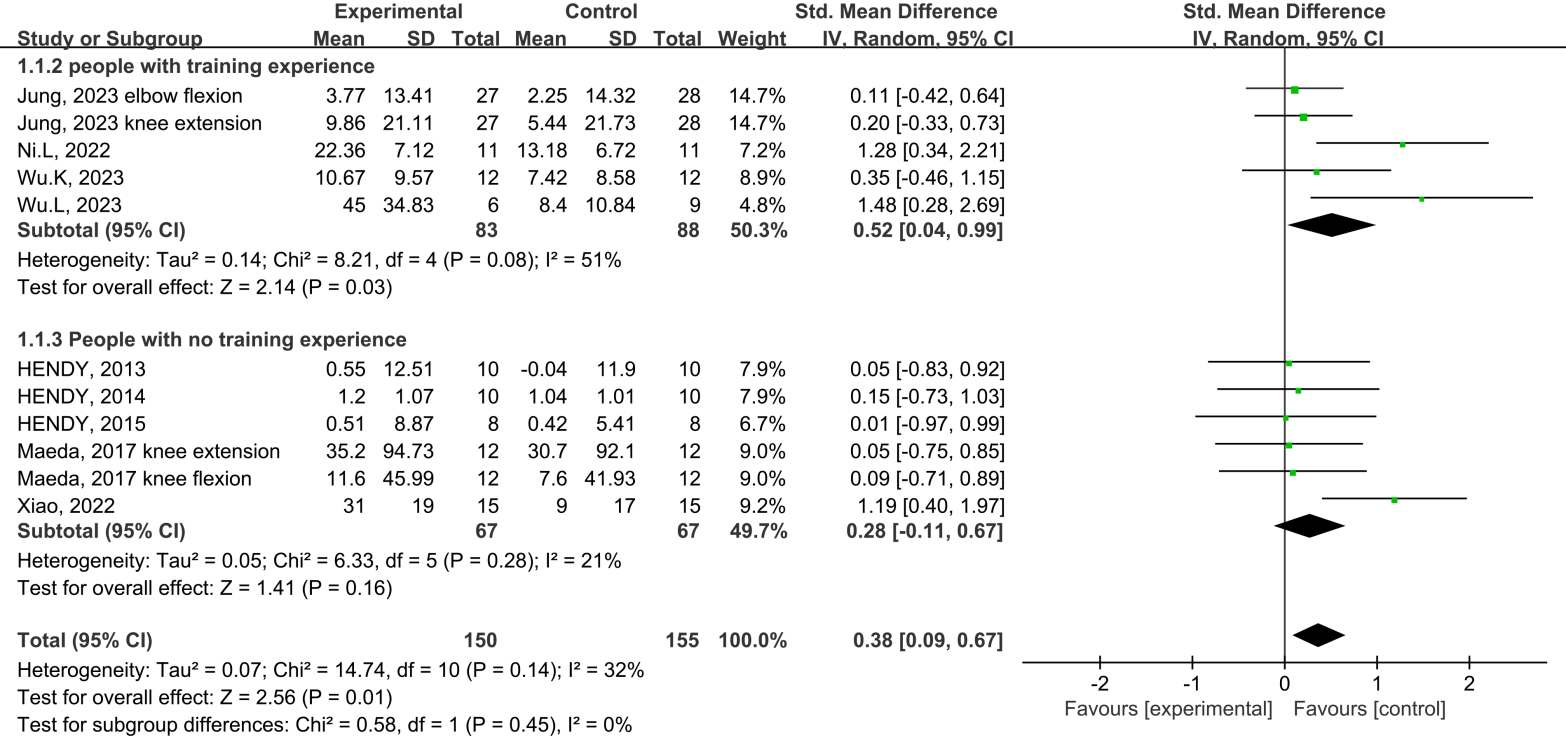
Subgroup analysis of the effects of tDCS combined with physical training intervention on muscle strength in people with and without training experience.

**Figure 6:**
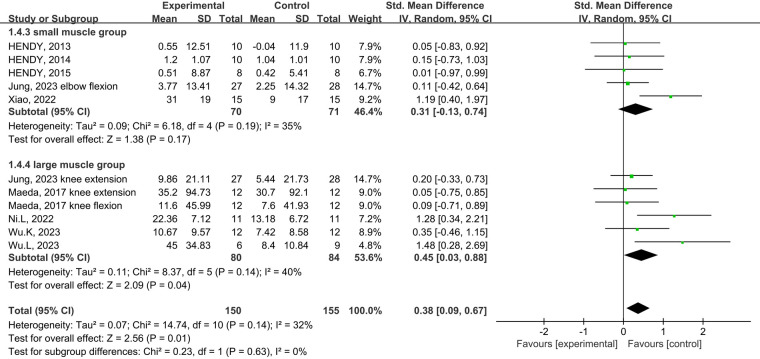
Subgroup analysis of the effects of tDCS combined with physical training intervention for different muscle groups on muscle strength.

Subgroup analysis of the subject population showed an increase in muscle strength in those with training experience following tDCS combined physical training (SMD = 0.39; 95% CI: 0.08, 0.70; *p *= 0.01), but not for those without training experience (SMD = 0.29; 95% CI: −0.06, 0.63; *p *= 0.10) (Figure 5). Subgroup analysis for muscle group showed muscle strength enhancement in large muscle group following tDCS combined physical training (SMD = 0.39; 95% CI: 0.08, 0.71; *p *= 0.01), but not for small muscle group (SMD = 0.29; 95% CI: −0.04, 0.63; *p *= 0.09) (Figure 6).

#### 3.4.2 Lower limb explosive strength

**Figure 7:**
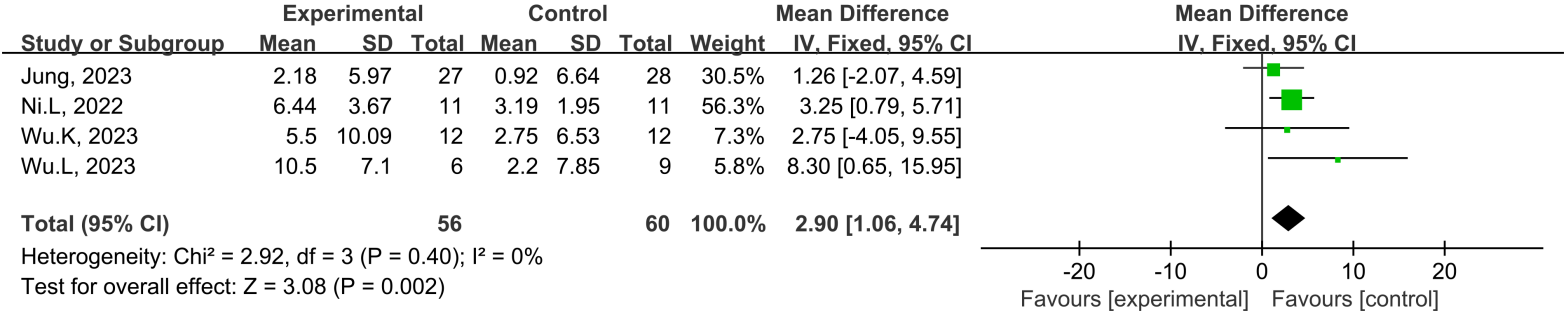
Meta-analyses of the effects of tDCS combined with physical training intervention on lower limb power.

A total of four studies from the included studies reported the effects of tDCS combined with physical training intervention on lower limb explosive strength. As the test for heterogeneity showed no heterogeneity (*p *= 0.40, *I*^2^ = 0%), and *I*^2^* *≥ 0%, we used the fixed-effects model for the effect size test. Based on the meta-analysis, tDCS combined with the physical training intervention was significantly more effective than physical training intervention alone in improving lower limb explosive strength, and the improvement of explosive strength reached the level of large effect size (MD = 2.90; 95% CI: 1.06, 4.74; *p *= 0.002) (Figure 7).

#### 3.4.3 Cortical excitability

**Figure 8:**
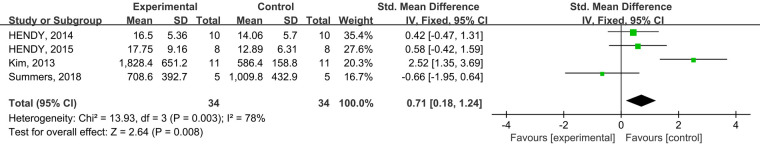
Meta-analyses of the effects of within-group tDCS combined with physical training intervention on cortical excitability.

**Figure 9:**
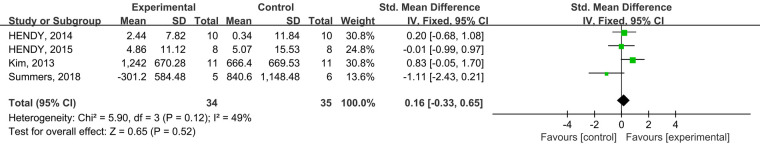
Meta-analyses of the effects of inter-group tDCS combined with physical training intervention on cortical excitability.

A total of four studies from the included studies reported the effects of tDCS in combination with physical training intervention on cortical excitability. Firstly, effect sizes were combined for within-group MEP wave amplitude, and the test for heterogeneity resulted in a low degree of heterogeneity (*p *= 0.003, *I*^2^* *= 32%), with *I*^2^* *≤ 50%, so we used the fixed-effects model for the effect size test. Secondly, effect sizes were combined for MEP wave amplitude between groups, and the heterogeneity test results were low degree of heterogeneity (*p *= 0.12, *I*^2^* *= 49%), and *I*^2^* *≤ 50%, so effect sizes were tested using the fixed effect model. Meta-analysis results showed that tDCS combined with physical training intervention, although it could increase cortical excitability (SMD = 0.71; 95% CI: 0.18, 1.24; *p *= 0.008) (Figure 8), but there was no statistical difference between the two groups compared to the control group (SMD = 0.16; 95% CI: −0.33, 0.65; *p *= 0.52) (Figure 9).

## 4 Discussion

This is the first systematic review explored the effects of physical training combined with tDCS on measures of muscle strength and cortical excitability. Results indicate that when physical training combined with tDCS, maximal strength and lower limb explosive strength could be significantly improved. Moreover, our results showed that there was a significant increase in the amplitude of MEP following the stimulation of the primary motor area of cerebral cortex with either tDCS combined with physical training or sham tDCS combined with physical training.

Muscle strength is influenced not only by myogenic factors but also by neural factors. The highest level structure controlling human movement is the motor area of the cerebral cortex, including the primary motor cortex, premotor cortex and supplementary motor cortex. Among them, the primary motor cortex is the most important part of the motor system, and the motor control areas are arranged in reverse order, with those controlling the lower limbs at the top of the cerebral hemispheres and those controlling the face located near the central sulcus, whose motor commands are directed to the motor neurons and interneurons in the spinal cord via the corticospinal tracts or the cortical brainstem tracts, which in turn control the muscular activities of the limbs and trunk (32). The central nervous system regulates muscle strength by elevating motor unit discharge frequency and motor unit synchronization (33). Firstly, the higher the central nervous system excitability, the higher the neuron firing frequency, which causes the muscle to start the next contraction before the previous contraction has finished, and the muscle single contractions are superimposed, resulting in an increase in muscle contraction tension (34). Secondly, the stronger the excitability of the central nervous system, the higher the degree of synchronization of motor unit initiation, so that more neurons produce excitation at the same time, sending out higher frequency and more concentrated nerve impulses, so that some less excitable motor units are involved in muscle contraction, thus increasing muscle strength (35).

tDCS is used as a brain modulation technique to alter the excitability of the cerebral cortex by applying lower electrical currents to specific areas of the brain for a period of time (36). It is important to note that tDCS does not produce potentiation changes, but rather depolarizes cell membranes by current stimulation. From a molecular perspective, tDCS regulates the opening of ion channels through voltage. In the resting state, the extramembrane Na+ concentration is significantly higher than the intramembrane. Upon stimulation, the Na+ channels are opened, allowing the ions to enter the intracellular membrane along the concentration gradient. Once the concentration reaches a certain threshold, a transient positive potential is generated (37). Meanwhile, it has also been shown that tDCS can change neuronal non-synaptic excitability by altering the intracellular Ca+ concentration in the brain, which in turn improves central nerve impulses (38). Some researchers have monitored changes in cortical excitability, and the results showed an increase in MEP wave amplitude and cortical excitation following tDCS (39). Increased cortical excitability allows more motor neurons to be involved in excitation, which increases muscle recruitment. In addition, tDCS can also indirectly improve brain function by increasing cerebral blood flow (40), modulating synaptic plasticity (41), and neural network connectivity (42), which in turn alters the excitability of spinal motor neurons and improves muscle strength.

In terms of improving muscle strength, it has been shown in many studies a single tDCS can result in an acute improvement in muscle strength. Kenville et al. (43) showed a significant increase in maximal isometric squat strength after a single tDCS in 25 healthy adults. Anoushiravani et al. (44) came to the same conclusions. However, these studies only explored the effect of a single acute tDCS on muscle strength and did not include combined tDCS physical training as an intervention. It is important to note that strength training can also alter brain function. It has been shown that after training at a certain intensity, cortical MEP amplitude increases (45), accompanied by a decrease in short-interval intracortical inhibition (SICI) (46). tDCS has a similar effect on changes in brain function, and when the two are combined, it is unclear whether the effects on the brain and even motor performance are enhanced or counteracted when the two are combined. Based on this some researchers have combined tDCS stimulation with physical training to investigate its effects on muscle strength, however the results have been mixed. In the present study, relevant literature was pooled and meta-analysed, and according to the results, tDCS in combination with physical training intervention improved muscle strength more than physical training intervention alone. Wu Li (29) showed that tDCS combined with weighted semi-squat jump training was effective in improving muscle strength in healthy college students. Xiao et al. (26) demonstrated that after four weeks of tDCS in combination with foot core strength training, metatarsal flexor strength as well as improvement of proprioceptive function of the ankle in healthy populations could be significantly improved. It is thought that this phenomenon is related to alterations in synaptic plasticity (47, 48). Generally, tDCS induces immediate effects by changing neurons’ resting membrane potentials, which usually dissipate after an hour (49). In this study, which is indexed by the power of selection, the duration of tDCS stimulation was more than two weeks. tDCS repetitive stimulation provides a cumulative and more lasting effect (25), which is closely related to the rise in the expression of N-methyl-D-aspartate (NMDA) and *α*-amino-3-hydroxy-5-methyl-4-isoxazolepropionic acid (AMPA) at glutamate-sensitive receptors in the postsynaptic membrane relationship, with elevated NMDA and AMPA expression increasing sensitivity to glutamate thereby triggering postsynaptic potentials (50). In addition, synaptic efficacy may also be affected by changes in astrocytic calcium ion concentrations (51).

However, muscle strength after tDCS stimulation in combination with physical training interventions did not differ significantly between groups, but pre- and pos*t*-tests within the groups changed significantly. A study by Jung et al. (15) found that the maximum isometric strength of the elbow flexors and knee extensors in the experimental group changed significantly after tDCS combined with physical training interventions as compared to the baseline level, while at the same time, the muscle strength in the control group also increased. So the increase in muscle strength between the groups was not statistically significant. Maeda et al. (17) performed centrifugal contraction training of knee extension and knee flexion with combined tDCS stimulation on 24 healthy adults for 3 weeks, and results showed that both experimental and control groups increased muscle strength in knee extension and knee flexion, but that there was no significant difference between groups in muscle strength. It has been explained that the slightly longer interval between tDCS stimulation three times per week was not enough to affect synaptic plasticity in the motor cortex, and that increasing the number of tDCS interventions per week might have a better effect (15, 52). Liu et al. (53) administered tDCS intervention five times per week to professional rowers, and the athletic performance significantly improved after two weeks of the intervention. Similarly it has been demonstrated that daily tDCS stimulation is more likely to alter cortical excitability than spaced stimulation (52). This may be attributed to a direct enhancement of protein synthesis by tDCS during the training period, or alternatively, to a downstream interaction of its excitatory effects with exercise-related protein synthesis during and after the training period (54).

Subgroup analyses found that tDCS in combination with large muscle group training improved muscle strength more than small muscle groups. Hendy and Kidgell (25) performed tDCS stimulation combined with weighted dumbbell wrist extension training on 30 participants over a three-week period, and the results showed that neither experimental nor control groups showed significantly different changes in muscle strength. Similar results have also been obtained by combining tDCS stimulation with biceps dumbbell curl training. However, when tDCS stimulation was combined with large muscle group training, the training effect of the combined intervention was significantly better than that of the training group alone (16, 17, 30). These results suggest that training of different muscle groups may influence the stimulation effect of tDCS. It is possible that compared to small muscles, large muscles possess more potential to improve muscle strength (55). Specifically, Larger muscles possess a larger cross-sectional area and contain a greater number of motor neurons than smaller muscles. However, not all motor neurons are recruited during muscle contraction; rather, only a subset of them is engaged. Furthermore, the number of unrecruited neurons is greater in larger muscle groups than in smaller ones, suggesting that the potential for increased muscle strength is greater in the former (56). tDCS engages a greater number of unrecruited motor units in muscle contraction, resulting in a greater force output (37).

The results of another subgroup analysis showed that muscle strength was more likely to be improved after the tDCS combined physical training intervention in people with training experience compared to people without training experience. This may be due to the fact that people with training experience have better movement stability and high stability of the test movements when performing the pre and pos*t*-tests of the experiment due to regular training, i.e., there are fewer additional variables in the experiment and more net intervention efficacy induced by the experiment (57). However, it has also been shown that after tDCS training alone, there is a higher potential for improvement in muscle strength in diseased populations (58, 59) compared to healthy populations (60, 61) due to the fact that diseased populations such as stroke patients may have a greater potential for strength enhancement due to factors such as decreased cortical excitability, and blocked neural pathway conduction. Therefore, future research on tDCS could focus more on athletes or people with good athletic experiences to investigate whether the “ceiling” effect may affect performance enhancement.

The vertical jump is one of the most common indicators of lower limb explosive strength (62). In this study, the relevant literature was pooled and Meta-analyzed, and the results showed that tDCS in combination with physical training improved lower limb explosive strength in a healthy population more than physical training intervention alone. Ni (30) found a significant increase in vertical jump height after six-week tDCS stimulation in combination with squat training intervention in 33 healthy male college students (30). Many foreign studies have confirmed the significant effect of tDCS on lower limb explosive strength enhancement (44, 63, 64). tDCS combined with physical training intervention to improve the physiological mechanism of explosive strength is currently unknown. It has been shown that an increase in the frequency of spinal motor neuron impulse delivery at the beginning of muscle contraction is the main reason for the elevated rate of training-induced contraction force generation (RFD) (65). tDCS stimulation increases cortical excitability, which in turn increases the downward nerve impulses from cortical spinal tract fibers to control the activity of spinal motor neurons innervating skeletal muscles. Therefore, increasing the frequency of spinal motor neuron impulse delivery through the combined effect of tDCS stimulation as well as training may be one reason for inducing increased explosive power. Another reason could be due to the decrease in SICI that promotes muscle strength. Weier et al. (46) found that after several weeks of intense strength training, muscle strength increased while SICI continued to decrease. This is consistent with changes in SICI after tDCS stimulation (66). Additionally, a correlation between enhanced motor performance and increased corticospinal excitability and decreased SICI following tDCS intervention has been demonstrated (67). Hendy and Kidgell (16) concluded that SICI was significantly reduced only when strength exercise was combined with the tDCS condition. In addition to exploring the starting point from a neural perspective, it has also been hypothesised that tDCS increases explosive power output per second by improving the integration efficiency of the energy supply system (68).

Motion-evoked potential (MEP) is the potential recorded on the surface of the corresponding muscles or nerves when the excitation generated by the application of electrical or magnetic stimulation to cortical motor areas depolarizes the anterior horn cells of the spinal cord or peripheral neuromotor fibres through downward conduction pathways (69), and it is one of the most important indexes used to reflect the changes in the excitability of the corticospinal nerve bundles. Meta-analysis of the present study showed that there was no between-group difference in the effect of tDCS combined with physical training intervention on MEP amplitude, but MEP amplitude was significantly increased within the group.

After tDCS in combination with biceps curl training, Hendy et al. (28) reported the same findings, but the differences between groups were not statistically significant when compared to the physical training intervention group. This suggests that unilateral strength training-induced cortical plasticity combined with tDCS-induced plasticity has complementary effects on neuromodulation of motor pathways controlling the inactive limb. Kim et al. (27) found that not only was MEP wave amplitude significantly increased after tDCS combined with an physical training intervention in participants, but there was also a statistically significant between-group difference when compared to the control group. However, Summers et al. (19) study came up with the opposite result, after performing tDCS stimulation combined with finger tracking training on 14 participants, MEP amplitude decreased in the tDCS combined physical training intervention group while MEP amplitude increased in the physical training only intervention, probably because tDCS intervention along with strength training causes a disruptive effect on cortical excitability. As a result, fewer studies have examined tDCS's effects on cortical excitability in combination with physical training, and the results can differ considerably depending on the area stimulated and the training intervention. It would therefore be beneficial for future studies to examine the effects of tDCS in combination with physical training on the excitability of the cortex.

## 5 Limitations and prospects

Research on tDCS combined with physical training intervention has focused on healthy people, and few studies have focused on athletes. tDCS stimulation frequency and duration, as well as the program of the physical training will affect the final performance of the physical training, and there is almost no research in this area. Different tDCS stimulation devices have different effects on the results of the study. tDCS stimulation devices available on the market include the Halo sport, HD-tDCS, and sponge tDCS. It is suggested that future research on tDCS could be conducted from the perspectives of the study population, the program of the training, and the stimulation device.

(1) Study population: The selection of participants is not only limited to the healthy people, but also can be spread to athletes, to further explore whether the tDCS combined physical training intervention will have a “ceiling” effect due to prolonged training adaptations. (2) Training protocal: Different training contents and different load choices will have different effects on neural stimulation, and different weekly training frequencies will have different effects on the cerebral cortex, therefore, the content of the physical training interventions, the frequency of the training, and the selection of the load intensity are all issues that deserve to be further explored in depth. (3) Selection of the type of tDCS stimulation instrument: Different types of instruments have different strengths and weaknesses. Sponge electrode tDCS stimulation is less accurate and may induce changes in the excitability of neighboring areas of the cerebral cortex, making the results inaccurate. High-definition tDCS (HD-tDCS) can accurately localise the corresponding areas of the cerebral cortex, and the stimulation range is more precise. Halo sport is more convenient to wear than the former two, but the stimulation accuracy may be lower. Therefore, the choice of tDCS stimulation devices can be diversified in the future.

## 6 Conclusions

Compared with conventional physical training, tDCS combined with physical training intervention can more effectively improve muscle strength, lower limb explosive power. More specifically, compared to tDCS combined with training of small muscle groups, tDCS combined with training of large muscle groups was more effective in improving muscle strength. People with training experience are more likely to improve muscle strength after tDCS than those without training experience. In addition, the combination of tDCS with physical training and sham tDCS with physical training both resulted in increased cortical excitability.

## Data Availability

The original contributions presented in the study are included in the article/Supplementary Material, further inquiries can be directed to the corresponding author.
